# Rab11a Regulates the Development of Cilia and Establishment of Planar Cell Polarity in Mammalian Vestibular Hair Cells

**DOI:** 10.3389/fnmol.2021.762916

**Published:** 2021-11-19

**Authors:** Bin-Jun Chen, Xiao-qing Qian, Xiao-yu Yang, Tao Jiang, Yan-mei Wang, Ji-han Lyu, Fang-lu Chi, Ping Chen, Dong-dong Ren

**Affiliations:** ^1^Department of Otorhinolaryngology, ENT Institute, Eye and ENT Hospital, Fudan University, Shanghai, China; ^2^NHC Key Laboratory of Hearing Medicine, Fudan University, Shanghai, China; ^3^Shanghai Auditory Medical Center, Shanghai, China; ^4^Department of Cell Biology, Emory University, Atlanta, GA, United States; ^5^Department of Otolaryngology, Emory University, Atlanta, GA, United States

**Keywords:** Rab11a, cilia, planar cell polarity, vestibular, development

## Abstract

Vestibular organs have unique planar cell polarity (Figure 1A), and their normal development and function are dependent on the regular polarity of cilia (Figure 1B) requires. Rab11a is a small G protein that participates in the transportation of intracellular and extracellular materials required for polarity formation; however, our understanding of the mechanisms of the actions of Rab11a in vestibular organs is limited. Here, we showed that the general shape of the utricle was abnormal in *Rab11a*^*CKO/CKO*^ mice. These mice also showed abnormal morphology of the stereocilia bundles, which were reduced in both length and number, as well as disturbed tissue-level polarity. Rab11a affected the distribution of polarity proteins in the vestibular organs, indicating that the normal development of cilia requires Rab11a and intraflagellar transportation. Furthermore, small G protein migration works together with intraflagellar transportation in the normal development of cilia.

**FIGURE 1:**
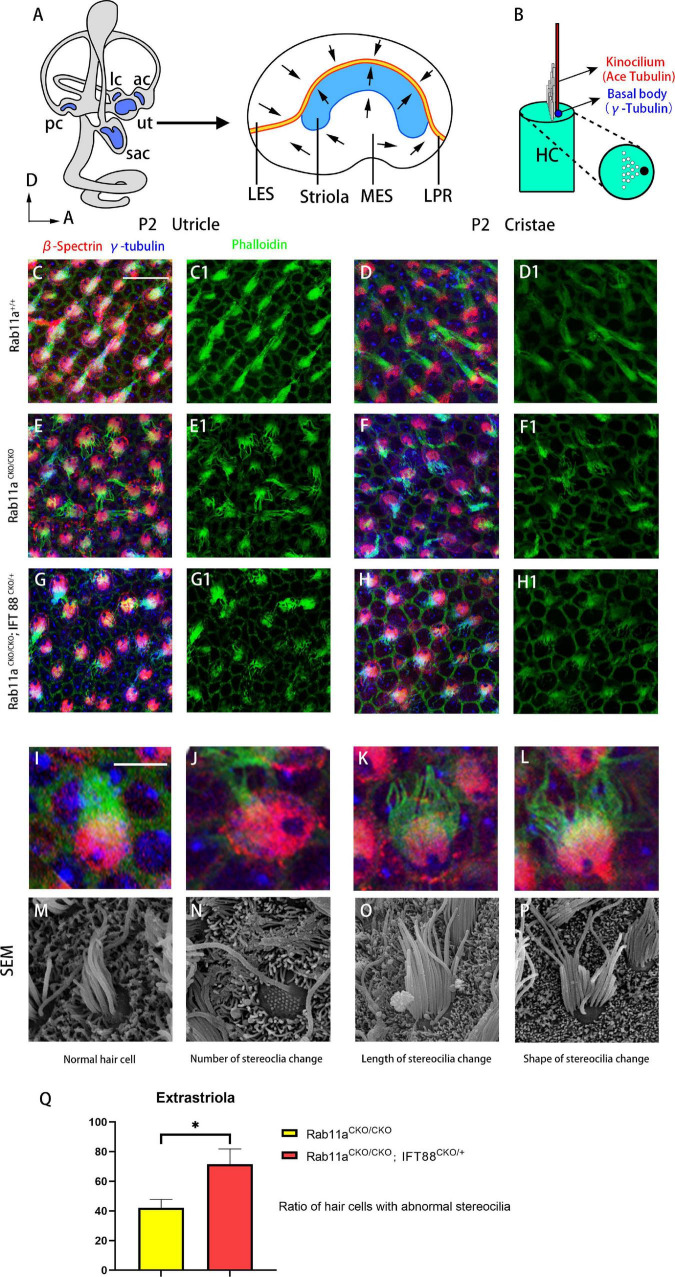
Morphological changes of stereocilia in the extrastriolar hair cells from *Rab11a* single or *Rab11a*/*IFT88* double-mutant utricles. **(A)** Medial view of a mouse left inner ear with its five vestibular sensory organs (gray). Enlarged are the utricle showing their subdivisions, LPR (yellow line), and striola (blue). LES, lateral extrastriola; MES, medial extrastriola; LPR, line of polarity reversal. **(B)** Schematic view of vestibular hair cell. Kinocilium is marked with ace-tubulin. Basal body is marked with γ-tubulin. **(C,C1,D,D1)** Normal appearance of the stereocilia of extrastriolar hair cells of wild-type controls. **(E,E1,F,F1)** Altered morphology in *Rab11a*^*CKO/CKO*^ animals. **(G,G1,H,H1)** The changes in the stereocilia morphology were more severe in *Rab11a*^*CKO/CKO*^/*IFT 88^*CKO/+*^* mice. **(I–L)** Higher magnification of confocal images of hair cells. **(M–P)** Scanning electron microscopy images of hair cells from wild-type controls and *Rab11a* mutants. **(I,M)** Morphology of normal. hair cells of wild-type controls. **(J,N)** The number of stereocilia on a single hair cell was deceased in the *Rab11a* mutant. **(K,O)** Stereocilia were shorter in mutants compared to the wild-type controls. **(L,P)** The staircase-like hair bundle architecture of hair cells was lost in *Rab11a* mutant mice. **(Q)** The percentage of hair cells with abnormal development of static cilia bundles in the extrastriola region was counted as a percentage of the total (*n* = 5). The percentage of abnormal hair cells was higher in Rab11a*^*CKO/CKO*^*, IFT88*^*CKO/+*^* mice compared to Rab11a*^*CKO/CKO*^*. The abnormal ratios of single and double knockout hair cells were 42.1 ± 5.7 and 71.5 ± 10.4, respectively. In **(A–J)**, for all primary panels, hair cell stereociliary bundles were marked with phalloidin (green), the actin-rich cuticular plate of hair cells was labeled with β-spectrin (red), while the basal body of the hair cell was labeled with γ-tubulin (blue). Scale bars: 10 μm **(C–H1)**, 5 μm **(J–N)**. *P < 0.05.

Morphological changes of stereocilia in the extrastriolar hair cells from *Rab11a* single or *Rab11a*/*IFT88* double-mutant utricles. **(A)** Medial view of a mouse left inner ear with its five vestibular sensory organs (gray). Enlarged are the utricle showing their subdivisions, LPR (yellow line), and striola (blue). LES, lateral extrastriola; MES, medial extrastriola; LPR, line of polarity reversal. **(B)** Schematic view of vestibular hair cell. Kinocilium is marked with ace-tubulin. Basal body is marked with γ-tubulin. **(C,C1,D,D1)** Normal appearance of the stereocilia of extrastriolar hair cells of wild-type controls. **(E,E1,F,F1)** Altered morphology in *Rab11a*^*CKO/CKO*^ animals. **(G,G1,H,H1)** The changes in the stereocilia morphology were more severe in *Rab11a*^*CKO/CKO*^/*IFT 88^*CKO/+*^* mice. **(I–L)** Higher magnification of confocal images of hair cells. **(M–P)** Scanning electron microscopy images of hair cells from wild-type controls and *Rab11a* mutants. **(I,M)** Morphology of normal. hair cells of wild-type controls. **(J,N)** The number of stereocilia on a single hair cell was deceased in the *Rab11a* mutant. **(K,O)** Stereocilia were shorter in mutants compared to the wild-type controls. **(L,P)** The staircase-like hair bundle architecture of hair cells was lost in *Rab11a* mutant mice. **(Q)** The percentage of hair cells with abnormal development of static cilia bundles in the extrastriola region was counted as a percentage of the total (*n* = 5). The percentage of abnormal hair cells was higher in Rab11a*^*CKO/CKO*^*, IFT88*^*CKO/+*^* mice compared to Rab11a*^*CKO/CKO*^*. The abnormal ratios of single and double knockout hair cells were 42.1 ± 5.7 and 71.5 ± 10.4, respectively. In **(A–J)**, for all primary panels, hair cell stereociliary bundles were marked with phalloidin (green), the actin-rich cuticular plate of hair cells was labeled with β-spectrin (red), while the basal body of the hair cell was labeled with γ-tubulin (blue). Scale bars: 10 μm **(C–H1)**, 5 μm **(J–N)**. *P < 0.05.

## Introduction

The morphology and polarization of hair cells in the vestibular organs are essential for maintaining balance and sensing head movement. In mammals, the vestibular system is composed of five sensory organs within the inner ear: the utricle, the saccule, and three ampullae (Cullen, 2019). The utricle and saccule sense linear acceleration, while the ampullae located at the end of the semicircular canal are responsible for detecting rotational acceleration (Lempert et al., 1998; Wang and Nathans, 2007). Detecting and transducing mechanical signals from the environment to electrical signals in neurons are dependent on the well-aligned cilia bundles on the hair cells (Fettiplace and Kim, 2014). The hair bundle is composed of a kinocilium with a microtubule core, and stereocilia that develop from microvilli. The kinocilium becomes asymmetrically aligned during development, leading to the formation of staircase-aligned stereocilia from behind. This asymmetrical location of hair bundles on the planar plane of the hair cells is defined as hair cell polarity (Furness and Hackney, 2006; Fettiplace and Kim, 2014). Intact hair cell polarity is indispensable for hearing (Jacobson et al., 2008) and maintaining balance in three-dimensional space (Ren et al., 2013). Furthermore, hair cells in each sensory organ of the inner ear are arranged in a coordinated manner that manifests as a specific pattern of planar cell polarity (PCP) (Rida and Chen, 2009). The hair cells in the vestibular organ are highly organized, with a precise microstructure that is important for balance function. Vestibular hair bundles contain 50–100 actin-filled stereocilia, arranged in 10–15 ranks of successively increasing height, giving the bundle a staircase-like appearance. During embryonic development, the PCP signaling pathway can lead cells to convergent extension and oriented cell division, leading to organ morphogenesis. Mutation of the PCP gene can cause serious developmental defects, including neural tube defects, cystic kidney disease (Karner et al., 2009), bone disease (Randall et al., 2012), and congenital heart malformations (Gibbs et al., 2016).

The asymmetric distribution of planar cell polar proteins is a characteristic of polarity. Gαi3 plays an important role in the asymmetric distribution of stereocilia. The Insc/Gαi/LGN complex is formed in the bare zone near the outer side that lacks microvilli, and the aPKC/Par3/Par6 complex is found in the opposite direction near the middle (Ezan et al., 2013; Tarchini et al., 2013). Gαi3 and LGN are also distributed at the top of the stereocilia bundles adjacent to the bare area (Tarchini et al., 2016).

The Rab GTPases are key regulators of intracellular membrane trafficking and endocytic recycling. Rab11 is a small G protein belonging to the Ras superfamily, which plays an important role in regulating the expression of cell surface receptors and adhesion proteins. It has been reported that members of the Rab protein family are related to ciliary transportation, and Rab11 is involved in the formation of protein complexes and in endocytosis and exocytosis (D’Souza-Schorey and Chavrier, 2006; Bos et al., 2007; Stenmark, 2009). In retinoblasts *in vitro*, Rab11a interacted with Rab GTPases in intracellular transportation during the formation of cilia (Knödler et al., 2010). Rab11 was also reported to interact with the core PCP protein, Vangl2. The expression of Rab11 was affected by Vangl2 expression and affected the distribution of Vangl2 during gastrula formation in *Xenopus laevis* (Ossipova et al., 2015).

However, whether Rab protein has a regulatory effect on mammalian inner ear cilia development remains unknown. The mammalian genome encodes three Rab11 proteins, designated as Rab11a, Rab11b, and Rab11c. We found that Rab11a is expressed in the basal body (Supplementary Figure 1) of the vestibular organs and plays an important role in cilia formation via the interaction of intraflagellar transport (IFT), the PCP, and tissue polarity.

## Materials and Methods

### Mouse Strains and Animal Care

Animal care and use were performed in accordance with the National Institutes of Health Guide for the Care and Use of Laboratory Animals and the experiments received approval from the Emory University Institutional Animal Care and Use Committee. *Rab11a* conditional knockout mice and *IFT88* conditional knockout mice were described previously (Kibar et al., 2001; Haycraft et al., 2007; Yu et al., 2014). The Looptail mouse strain with a missense mutation in *Vangl2* was obtained from The Jackson Laboratory (Jax stock #000220; Bar Harbor, ME, United States). *Rab11a* and *IFT88* conditional alleles were inactivated via Cre recombinase in the *Foxg1*^*Cre*+^ mouse line as described previously (Hébert and McConnell, 2000; Pirvola et al., 2002). In this article, *Rab11a*^*CKO/CKO*^ indicates that Rab11a has been knocked out via *Foxg1*^*Cre+*^. Similarly, *Ift88*^*CKO/CKO*^ indicates that Ift88 has been knocked out via *Foxg1*^*Cre+*^. All Rab11a^+/+^ mice in this research are littermates of mutants such as Rab11a^*CKO/CKO*^ mice.

### Whole-Mount Immunostaining

Neonatal (P0) or postnatal day 2 (P2) *C57Bl/6* mice were anesthetized on ice and disinfected with 75% alcohol and AnEr iodine. The heads of mice were removed and placed in a glass petri dish with sterile 1 × phosphate-buffered saline (PBS). The skull and brain were removed to expose the otic vesicles before fixing with 4% paraformaldehyde in PBS for 1 h at room temperature. The otic vesicles were washed with 1 × PBS three times and then stored at 4°C.

The freshly dissected otic vesicles were placed into sterile 1 × PBS. Microdissecting forceps were used to enter from the cochlea and the inner wall of the vestibule. The bone outside was removed to expose the utricle and saccule, and then the upper cap membrane was peeled off and the otoliths were cleaned carefully to obtain only the vestibular sensory epithelia.

The vestibular epithelia were incubated in blocking solution consisting of 10% donkey serum and in PBS-T (0.1% Triton X-100 in 1 × PBS) at room temperature for 1 h, followed by incubation with primary antibody in PBS-T supplemented with 5% donkey serum overnight at 4°C. After washing three times in PBS-T for 1 h each time, tissues were incubated in a solution containing secondary antibody with or without phalloidin in PBS-T supplemented with 5% donkey serum for 2 h at room temperature. Tissues were again washed three times in PBS-T, mounted in Fluoromount-G (#0100-01; SouthernBiotech, Birmingham, AL, United States) covered with 1.5-cm coverslips, and sealed.

The following primary antibodies were used: Rab11a (#2413, 1:200; Cell Signaling Technology, Danvers, MA, United States), LGN (1:200, a gift from Fumio Matsuzaki, RIKEN, Kobe, Japan) (Konno et al., 2008), β-spectrin (#612562, 1:200; BD Transduction Laboratories, Franklin Lakes, NJ, United States), acetylated tubulin (#23950, 1:400; Santa Cruz Biotechnology, Dallas, TX, United States), Prickle2 (1:500; a gift from Doris K Wu, NIDCD Laboratory of Molecular Biology, Bethesda, MD, United States), g-tubulin (#T6657, 1:200; Sigma Aldrich, St. Louis, MO, United States), and Vangl2 (#AF4815, 1:200; R&D Systems, Minneapolis, MN, United States).

The following secondary antibodies were used: donkey anti-rabbit-647 (#2492288, 1:1,000; The Jackson Laboratory), donkey anti-rabbit-555 (#2307443, 1:1,000; The Jackson Laboratory), donkey anti-mouse-647 (#2340862, 1:1,000; The Jackson Laboratory), donkey anti-mouse-555 (#2340813, 1:1,000; The Jackson Laboratory), donkey anti-sheep-488 (#2340750, 1:1,000; The Jackson Laboratory), FITC-conjugated phalloidin (#A12379, 1:1,000, Alexa Fluor; Thermo Fisher Scientific, Waltham, MA, United States), and Rho-conjugated phalloidin (#R415, 1:200, Alexa Fluor; Thermo Fisher Scientific).

The specimens were first pre-screened with an Olympus IX71 inverted fluorescence microscope. Confocal images were obtained with either a Leica TCS SP8 or Zeiss LSM510 with excitation wavelengths of 488, 543, and 633 nm.

### Scanning Electron Microscopy

Epithelia were washed three times in PBS for 10 min each time, and then fixed in 2.5% glutaraldehyde in 0.1 M PBS at room temperature for 30 min. Samples were then rinsed with 0.1 M cacodylate buffer followed by post-fixation with 1% osmium tetroxide in 0.1 M cacodylate for 1 h. Next, the samples were sequentially fixed in 30 and 50% ethanol for 15 min each, and 70% ethanol overnight at 4°C. The next day, the samples were fixed in 80, 90, and 100% ethanol for 15 min each. The specimens were placed into labeled microporous capsules and loaded into the sample boat of a chilled Polaron E3000 critical point drying unit (Quorum Tech, Laughton, United Kingdom). Samples were sputtered with gold for 3.5 min and scanned at 10 kV using a field emission scanning electron microscope (DS-130F; Topcon, Tokyo, Japan).

### Phenotypic and Statistical Analysis

Pictures were processed using Adobe Photoshop CC2017 (San Jose, CA, United States). Statistical analyses of relevant angle data were performed using SPSS ver. 22 (IBM Corp., Armonk, NY, United States). Differences in the means between groups were analyzed using the *t*-test. In all analyses, *P* < 0.05 was taken to indicate statistical significance. GraphPad Prism6 (San Diego, CA, United States) was used to draw histograms. The rose function in MatLab software (MathWorks, Natick, MA, United States) was used to draw rose diagrams to show the polar distribution of utricle hair cells.

## Results

### *Rab11a*^*CKO/CKO*^ Leads to Morphological Changes in Cilia in the Mouse Vestibule

#### Morphological Changes of Stereocilia in the *Rab11a* Mutant Utricle

The morphology of the stereocilia bundles was disrupted in the *Rab11a*^*CKO/CKO*^ utricle and cristae, while the kinocilium was morphologically normal. Immunofluorescence staining revealed disordered arrangement and dispersion of stereocilia in the utricle and cristae (Figures 1C–F1). In the extrastriolar area, stereocilia of wild-type mice were arranged in clusters behind the kinocilium. In contrast, the stereocilia bundles showed morphological alterations in their length, number, and stepped arrangement in the *Rab11a*^*CKO/CKO*^ utricle. The percentage of hair cells with abnormal development of stereocilia tracts in the extrastriolar area was determined (in five areas in each sample). The proportion of abnormal hair cells was higher in *Rab11a*^*CKO/CKO*^ mice than in wild-type controls with a ratio of 42.1 ± 5.7 (Figure 1Q).

Given that the intraflagellar transporter IFT88 was reported to affect the development of the cochlear PCP, we speculated that Rab11a, as a component of the transportation system, may affect cilia formation via the interaction with IFT88. We used *IFT88* and *Rab11a* double mutants to examine the mechanism underlying the role of Rab11a in vestibular development. As conditional double knockout of *Rab11a* and *IFT88* is lethal, we used *Foxg1^*Cre+*^; IFT88^*fl/+*^; Rab11a^*fl/fl*^* mice to screen for polarity phenotypes. In the utricle and cristae, the changes in the stereocilia were more severe and more stereocilia bundles were lost in these mutants compared to *Rab11a* single mutants (Figures 1G-H1). The abnormalities of the stereocilia, including the number, length, and shape of the stereocilia, could be seen by scanning electron microscopy (Figures 1I–P). The proportion of abnormal hair cells in *Rab11a^*CKO/CKO*^; IFT88^*CKO/+*^* mice was higher compared to *Rab11a*^*CKO/CKO*^ mice. The abnormal ratios of single- and double-knockout hair cells were 42.1 ± 5.7 and 71.5 ± 10.4, respectively (Figure 1Q).

#### Kinocilium Shortening or Loss in *Rab11a* and Intraflagellar Transport Double-Mutant Mice

The PCP of vestibular organs consists of three levels: subcellular, intercellular, and tissue-level polarity (Deans, 2013). The kinocilium is tethered to the tallest rod of the hair bundle. The stereocilia staircase is built next to the kinocilium after acquiring its final position (Lu and Sipe, 2016). Within a hair cell, cellular structures, such as the stereocilia, are positioned asymmetrically on the apical plane along the tissue axis, which is defined as subcellular polarity. Therefore, we used phalloidin staining to label the actin-enriched stereocilia, and acetylated tubulin to mark the kinocilia.

**FIGURE 2:**
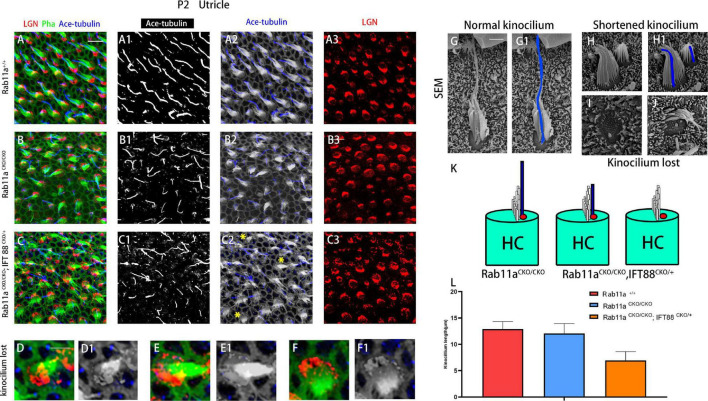
Kinocilia were shortened or lost in *Rab11a* IFT mutant mice. **(A–A3)** In P2 wild-type mice, kinocilia were located on one side of the hair cells, marking the planar cell polarity of single hair cells. **(B–B3)** The length and number of kinocilia were generally normal in *Rab11a* mutant utricles. **(C–C3)** Kinocilia were lost or shortened in *Rab11a*^*CKO/CKO*^; *IFT88*^*CKO/+*^ utricle (yellow asterisks). **(D–F1)** Enlarged images of hair cell marked with yellow arrowheads in **(C2)**. **(G,G1)** Scanning electron microscopy image of a normal hair cell. The kinocilia are outlined (blue). **(H,H1)** Shortened kinocilia were found in the hair cells of *Rab11a* mutant utricular macules. **(I,J)** The whole kinocilia were lost in the hair cells of *Rab11a* mutant utricular macules. **(K)** The schematic view of kinocilium shortened or lost in *Rab11a*^*CKO/CKO*^; *IFT88*^*CKO/+*^ utricle. **(L)** The average kinocilium length of control, *Rab11a*^*CKO/CKO*^, *Rab11a*^*CKO/CKO*^; *IFT88*^*CKO/+*^ utricle. The kinocilium length of control, single and double knockout hair cells were 12.90 ± 1.43, 12.07 ± 1.87, and 6.95 ± 1.67 μm, respectively. In **(A–F)**, for all primary panels, hair cell stereociliary bundles were marked with phalloidin (green), kinocilium were labeled with acetylated tubulin (blue), and intrinsic cell polarity was labeled with LGN (red). Scale bars: 10 μm **(A–C3)**, 5 μm **(D–F1)**, 1 μm **(G–J)**.

In the *Rab11a*^*CKO/CKO*^ mouse utricle, the length and number of kinocilia were generally normal compared to those of wild-type controls (Figures 2A–B3). The length and number of kinocilia were also reported previously to be unaffected in the utricles of *IFT88*^*CKO/CKO*^ mice (Jones et al., 2008). Furthermore, to characterize the hair bundle morphology of *Foxg1^*Cre+*^;Rab11a^*fl/fl*^; IFT88^*fl/+*^* mice, we applied both scanning electron microscopy and immunostaining (Figures 2C–J). The kinocilium lengths of wild-type, single-gene, and double-gene knockout hair cells were 12.9 ± 1.43, 12.1 ± 1.87, and 6.95 ± 1.67 nm, respectively. The kinocilium developed abnormally, and they became shorter or even disappeared (Figures 2K,L).

#### *Rab11a*^*CKO/CKO*^ Leads to Morphological and Line of Polarity Reversal Position Changes in the *Rab11a*^*CKO/CKO*^ Utricle

The Insc/Gαi/LGN complex guides the asymmetrical distribution of hair bundles. Therefore, we stained LGN to track the subcellular polarity of hair cells.

**FIGURE 3:**
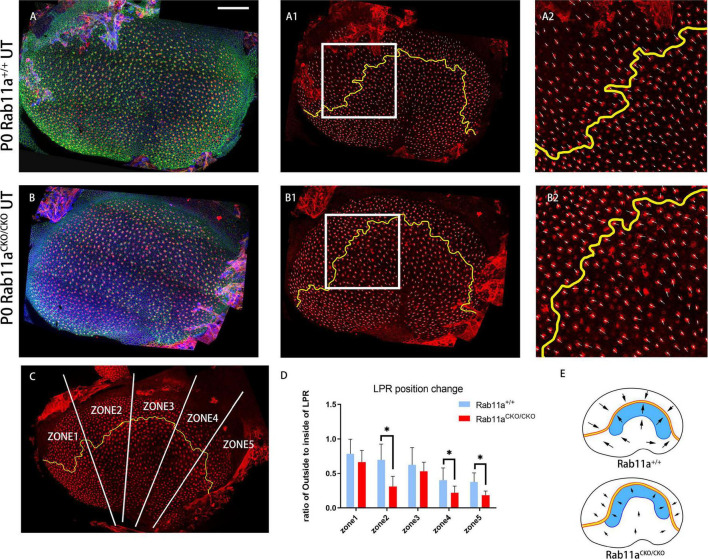
Changes in shape and position of the line of polarity reversal (LPR) in *Rab11a*^*CKO/CKO*^ utricles. **(A–A2)** In utricular macules of wild-type controls, the planar cell polarity of hair cells was labeled with the cell intrinsic planar cell polarity protein β-spectrin (red). White arrowhead shows the orientation of hair cells. Utricular hair cells were oriented with their bundles pointed toward each other along the LPR (yellow line). **(A2)** Zoomed image of white square in **(A1)**. **(B–B2)** In the *Rab11a* mutant utricle, the utricle was flattened compared to the wild-type utricle. **(B2)** Zoomed image of white square in **(B1)**. **(C)** The utricle was separated into five segments (white line) of similar size according to the length of the LPR (yellow line). The numbers of hair cells located inside and outside the LPR were counted. **(D)** The ratio of the number of hair cells located outside to inside of the LPR was calculated in each of the five segments for each genotype. Five mice were assayed at P0 for each of the wild-type and *Rab11a* knockout groups. Statistical analyses were performed using Student’s *t*-test with unequal variance, ^∗^*P* < 0.01. The lower ratios in regions 2, 4, and 5 of the *Rab11a*^*CKO/CKO*^ group indicated that there were fewer hair cells located outside the LPR compared to the wild-type controls, while the total number of hair cells in the utricle was not significantly different between the two groups, and we concluded that the LPR moved toward the outside. **(E)** Schematic view of control and Rab11a*^*CKO/CKO*^* utricle the LPR moved toward the outside in mutant utricle (black arrow). Scale bars: 100 μm.

In mouse vestibular organs, the subcellular polarity of hair cells is reflected by the direction and pattern of three-dimensional cilia bundles and the position of kinocilium. There was a lack of β-spectrin staining indicating the position of the basal body. The *Rab11a*^*CKO/CKO*^ mouse utricle had a flatter morphology compared to the controls. Furthermore, in the development of stereocilia bundles in the hair cells of *Rab11a^*CKO/CKO*^; IFT88^*CKO/*/+^* mice, the proportion of abnormally developed hair cells in the extrastriolar area was higher than that in *Rab11a*^*CKO/CKO*^ mice (Figure 1Q). Staining for the intrinsic polar protein LGN showed the direction of the hair cells (Figures 3A–B2), allowing us to draw the line of polarity reversal (LPR). To calculate the shift of LPR, we further counted the number of hair cells adjacent to the LPR (Figure 3C), which indicated that the LPR moved to the outside in *Rab11a*^*CKO/CKO*^ mice (Figures 3D,E).

#### Quantification of Vestibular Hair Cell Orientation in Control and *Rab11a* Mutant Utricles

The direction of hair cells in *Rab11a^*CKO/CKO*^; IFT88^*CKO/*/+^* utricles was disordered, while the stereocilia appeared to point neatly in the same direction in the normal utricle.

**FIGURE 4:**
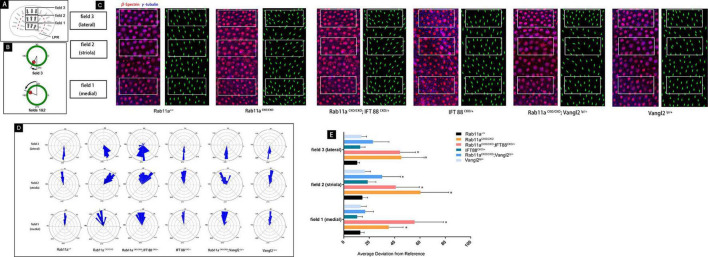
Quantification of vestibular hair cell orientation in control and *Rab11a* mutant utricles. **(A)** Schematic illustrating the positions of the three analysis fields relative to the position of the line of polarity reversal (LPR; red dashed line). Hair cells in the maculae are patterned about the LPR, and as a result cells in the lateral extrastriolar region (LES) have stereociliary bundle orientations that are opposite of those in the striola and medial extrastriolar region (MES). **(B)** Individual stereociliary bundle orientations for vestibular hair cells of the utricular maculae graphed as circular histograms indicated stereociliary bundle misorientation in the striolar region of the *Rab11a* mutant mice. A 90° is directed toward the lateral and 180° toward the medial edge of the utricular maculae, and each bin is 12°. The total numbers of hair cells represented by each histogram (*n*) are shown, and black bars mark the mean stereociliary bundle orientation. The dimensions of the analysis fields are all 100 mm × 50 mm. **(C)** Wild-type utricle hair cells labeled for planar polarity analysis with antibodies to γ-tubulin (blue) and β-spectrin (red). The region close to the LPR was selected from each sample. Individual hair cell orientations have been annotated based on the labeling. Utricles from *Rab11a*^*CKO/CKO*^ and *Rab11a^*CKO/CKO*^; IFT 88^*CKO/+*^* mice had misoriented hair cells throughout fields 1–3, while misoriented hair cells in utricles from *Rab11a^*CKO/CKO*^; Vangl2^*Lp/+*^* mice were restricted to field 2. **(D)** The average mean deviation of stereocilia bundle orientations was determined by measuring the absolute value of the angle formed by the bundle axis and a reference line drawn perpendicular to the striola. In this schematic, red indicates the position of the kinocilium and green is the cell periphery. **(E)** The average deviation from 0° for hair cells located in fields 1, 2, and 3 for each experimental and control genotype. Error bars indicate standard deviation. Statistical significance was calculated using Student’s *t*-test with unequal variance, ^∗^*P* < 0.01.

These results indicated that the *Rab11a* and *IFT88* gene was specifically knocked out in vestibular hair cells, the development of stereocilia was affected, and the polarity was also changed. To study the mechanism underlying the role of *Rab11a* in the core PCP pathway, we used *Rab11a^*CKO/CKO*^; Vangl2^*lp/+*^* mice and *Rab11a^*CKO/CKO*^; IFT88^*CKO/*/+^* double-knockout mice. First, we examined the direction of the hair cells to evaluate the changes in polarity of the tissues (Figures 4D,E). The direction of a single hair cell in the utricle could be determined by immunofluorescence analysis of the epidermal plate marker β-spectrin (Figure 4C). Studies have shown that Looptail mice with knockout of the *Vangl2* gene do not show effects on the direction of the hair cells in the plaques, while the hair cells of the semicircular canals are whorled (Qian et al., 2007). The hair cell orientation differed significantly in *Rab11a^*CKO*/*CKO*^; Vangl2^*lp/+*^* mice compared to wild-type mice. However, simply knocking out *IFT88* did not affect the hair cells in the vestibule. In *Rab11a^*CKO/CKO*^; IFT88^*CKO/*/+^* mice, the direction of hair cells was disordered (Figure 4E).

#### Subcellular Distributions of Core Planar Cell Polarity Proteins Are Affected in *Rab11a* Mutant Utricles

**FIGURE 5:**
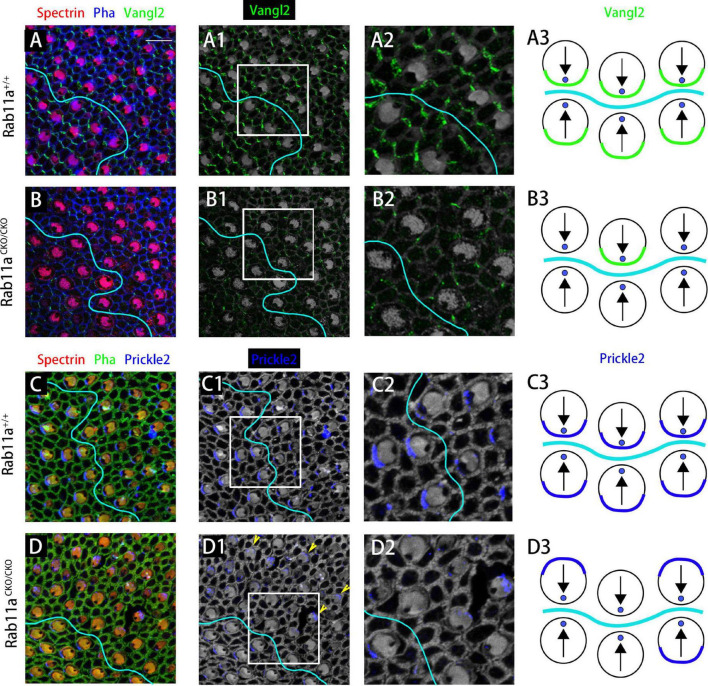
The subcellular distributions of core planar cell polarity (PCP) proteins are affected in *Rab11a* mutant utricles. **(A,A1,A2)** Vangl2 immunolabeling was enriched at cell boundaries in the wild-type utricular maculae. Asymmetrical protein localization was evident at many cell boundaries. **(B,B1,B2)** Vangl2 protein was significantly reduced from apical cell boundaries in *Rab11a*^*CKO/CKO*^ utricles. **(C,C1,C2)** Prickle2 was enriched at hair cell/support cell boundaries throughout the wild-type utricle. For cells located reversely across in different side of LPR, Prickle2 located in the same side of hair cells. **(D,D1,D2)** In *Rab11a*^*CKO/CKO*^ mice, the distribution of Pk2 changed in a region-specific manner. Prickle2 moved to the opposite side of hair cells located in two regions across the LPR (yellow arrowheads). **(A3,B3,C3,D3)** Schematic view of Vangl2 and Prickle2 expression in control and Rab11a*^*CKO/CKO*^* utricle. For primary panels in **(A–B2)**, hair cell stereociliary bundles were marked with phalloidin (blue), the actin-rich cuticular plate of hair cells was labeled with an antibody to β-spectrin (red), and with an antibody to the core PCP protein Vangl2 is shown in green. In **(C–D2)**, phalloidin is shown in green, β-spectrin in red, and core PCP protein Prickle2 in blue. Scale bars: 10 μm.

To study the PCP of adjacent cells, we performed immunostaining analysis for the core PCP proteins Vangl2 and Prickle2. In the wild-type utricle, Prickle2 and Vangl2 were distributed on the same side of the hair cell regardless of the side of the LPR. In the *Rab11a*^*CKO/CKO*^ utricle, the level of Vangl2 protein expression was significantly reduced compared to the wild-type controls (Figures 5A–B3). As no specific changes were observed in Vangl2 other than its expression level, we examined the expression of Prickle2 in the *Rab11a*^*CKO/CKO*^ utricle. Some hair cells in the utricles of *Rab11a*^*CKO/CKO*^ mice showed disordered distribution of Prickle2 compared with the controls (Figures 5C–D3), while the direction of the hair cells was still opposite to the two sides of the LPR. These observations confirmed that Rab11a, as an important transport tool, plays an important role in transporting polar proteins to the surface of hair cells, and its knockout affects the expression and distribution of polar proteins.

## Discussion

In the vestibular system, unlike the V-shaped arrangement of the cochlea, the stereocilia bundles of hair cells are clustered around the kinocilium. The appropriate arrangement of cilia is essential for hair cells to respond to stimuli and maintain balance function (Fettiplace and Kim, 2014).

The critical role of PCP core proteins in regulating planar polarization in various organs is well conserved across species. However, our understanding of the mechanisms underlying the actions of Rab11a in vestibular organs and how it regulates PCP core proteins is limited.

### Cilia Development

The kinocilium plays an important role in the development of hair cell cilia bundles. The extension and maintenance of the length of cilia relies on IFT to transport the required materials along the axons (Eatock and Songer, 2011). Specific knockout of IFT-related genes in the inner ear, including *IFT88*, *Kif3a*, and *IFT20*, leads to dysplasia or loss of kinocilia, and the stereocilia bundles become flattened (Jones et al., 2008; Sipe and Lu, 2011; May-Simera et al., 2015). Besides, deficiency of Ick/Cilk1, which encodes a ciliary kinase regulating IFT, results in kinocilia elongation and PCP defects including misshaping of stereocilia in the cochlea (Okamoto et al., 2017).

Knockout of *Rab11* in the retinoblast cells *in vitro* resulted in damage to the cilia without affecting the vestibular utricle hair cells, but the number, length, and shape of the stereocilia were altered.

Cilia are rich in actin, and the protein synthesis process does not occur in ciliated axons, but kinocilia are aggregated and maintained through IFT (Bisgrove and Yost, 2006; Davis et al., 2006). In the process of cilia transport, the cell transfers various substances to the anchor point through the IFT complex, of which IFT88 is a subunit. Studies of the inner ear of IFT88/Polaris mutants have shown that after conditionally knocking out the *IFT88* gene in cochlear hair cells, the position of the basal body changes. It is no longer polarized to the side of the cell but rather is present in the center of the hair cell. In some cells, kinocilia did not develop, the stereocilia were arranged in a circle, and the internal polarity of the cells was disrupted, while no morphological changes were observed in the vestibular hair cells (Jones et al., 2008).

The kinocilia of the utricle were missing or reduced in length in *Foxg1^*Cre+*^; Rab11a^*fl/fl*^; IFT88^*fl/+*^* mice. In addition, the phenotype of *Rab11a* and *IFT* mutant utricles included a higher proportion of abnormal cilia than the utricles of single *Rab11a* mutants. We concluded that the roles of Rab11a and IFT88 in the development of kinocilia are redundant, and their in stereocilia development partially overlap.

### Influence on Planar Cell Polarity

Disruption of the arrangement and polarity distribution of inner ear cilia bundles can cause hearing and balance disorders in mice and humans (Lempert et al., 1998; Littlewood Evans and Müller, 2000; Alagramam et al., 2001; Adato et al., 2005; Corey, 2009). In the inner ear sensory organs, the hair cells are arranged in a coordinated and regular manner, showing a unique PCP (Rida and Chen, 2009). The polar arrangement of hair cells plays an important role in the complete functioning of sensory organs (Jacobson et al., 2008). For the five vestibular organs, the relative arrangement of hair cells is indispensable for maintaining balance in three-dimensional space.

The PCP is reflected at three different anatomical levels in the vestibular organs. First, there is PCP at the intracellular level, the so-called intrinsic polarity of the cell. In mice, at about embryonic day 12.5, the hair cells begin to develop characteristics different from the precursor cells. A fibril appears in the center of the hair cell, and it is surrounded by micropili that develop into stereocilia. The original cilia gradually elongate into kinocilia, which is mediated by the core PCP protein. For example, core PCP protein migrates to the side of the hair cell, marking the establishment of the internal polarity of the cell (Deans, 2013). Second, there is intercellular polarity, marked by the localization of Dvl2/3, Fzd3, Pk, and Vangl2 that coordinate the arrangement of stereocilia between adjacent cells (Denman-Johnson and Forge, 1999). Our results showed that in the *Rab11a*^*CKO/CKO*^ utricle, the direction of the hair cells was disordered compared to the controls (Figure 4). In addition, immunofluorescence staining demonstrated changes in the expression of Vangl2 and Prickle-2 in the *Rab11a*^*CKO/CKO*^ utricle. Thus, Rab11a appears to affect the intercellular polarity by affecting Vangl2 and Prickle-2.

Finally, there is tissue polarity. In both the utricle and saccule, the LPR divides the sensory epithelial cells into two groups with opposite stereociliary bundle polarities that are able to detect movements in opposite directions (Deans, 2013). One hypothesis is that hair cells located on opposite sides of the LPR have unique transcriptional profiles and can respond to the PCP-based tissue polarity information in opposite manners. Emx2 is a candidate regulator for transcriptional patterning because the LPR did not form in Emx2 mutants (Holley et al., 2010). The mechanisms regulating tissue polarity and patterning of the LPR have not been elucidated. However, we observed abnormal LPR formation in the *Rab11a*^*CKO/CKO*^ utricle in the present study but did not investigate the underlying mechanisms.

## Conclusion

**FIGURE 6:**
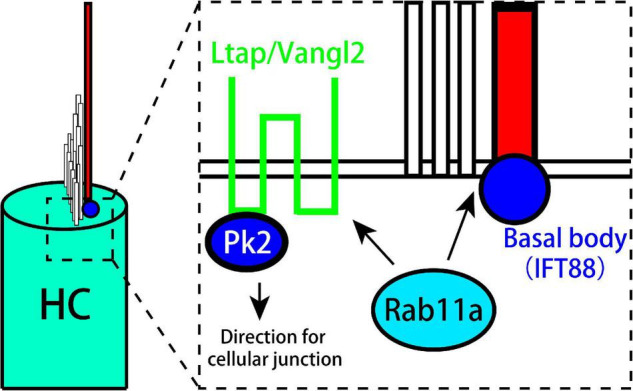
Model showing the role of Rab11a in the formation of planar cell polarity in utricle. Rab11a intracts with Ift88 expressed in kinocilium basal body to affect the development of vestibular cilia. Besides, Rab11a affects the polarization of PCP core proteins including Vangl2 and Prickle2.

Rab11a affects the development of cilia and the PCP of vestibular organs. In addition, it works together with IFT in the development of kinocilia and stereocilia. Rab11a also affects cell polarity by controlling the distribution of Vangl2 and Prickle-2, and can cause the LPR to move toward the outside to affect tissue polarity (Figure 6).

## Author’s Note

Planar cell polarity refers to the asymmetric distribution of cell morphology and polar proteins. The regular formation of planar cell polarity in vestibular organs is the basis for balance function. We found that small G protein knockout during development led to the disordered distribution of polarity proteins and immature phenotypes of sensory hair cells, suggesting its important role in vestibular organ development.

## Data Availability Statement

The original contributions presented in the study are included in the article/Supplementary Material, further inquiries can be directed to the corresponding authors.

## Ethics Statement

The animal study was reviewed and approved by the Institutional Animal Care and Use Committee Emory University.

## Author Contributions

D-dR, F-lC, and PC conceptualized and designed the study and reviewed the data. B-JC and X-qQ analyzed the data. B-JC, X-qQ, X-yY, and TJ wrote and edited the manuscript. Y-mW and J-hL retrieved and validated the data. D-dR and F-lC provided funding support, administered the project, and edited the manuscript. D-dR, F-lC, B-JC, X-qQ, and X-qQ provided data resources. All authors have reviewed, discussed and approved the manuscript.

## Conflict of Interest

The authors declare that the research was conducted in the absence of any commercial or financial relationships that could be construed as a potential conflict of interest.

## Publisher’s Note

All claims expressed in this article are solely those of the authors and do not necessarily represent those of their affiliated organizations, or those of the publisher, the editors and the reviewers. Any product that may be evaluated in this article, or claim that may be made by its manufacturer, is not guaranteed or endorsed by the publisher.

## Supplementary Material

Supplementary Figure 1 **(A)** Rab11a gene conditional knockout mice were constructed by inserting two Loxp sites between exons 2 and 5, and excising specific sheared exons 3,4 by binding to Cre recombinase. **(B)** Identification of genotype of Rab11a: the genotype of *Rab11*^*fl/fl*^ we need to obtain. Three cases can be seen in the figure. Pure type *Rab11*^*fl/fl*^ has a flox band with a product size of 564 bp. wild type *Rab11^+/+^* has a WT band with a product size of 400 bp. heterozygous Rab11a has both flox and WT bands. **(C)** Identification of genotype of Foxg1Cre: To specifically knock out Rab11a in the inner ear, *Foxg1*^*Cre*+^ was selected for specific expression. Cre-positive had a band and the product size was 500 bp.

Supplementary Figure 2 Rab11a expression in hair cells of the wild-type mouse vestibular system. In the P0 utricle **(A–A3)** and saccule **(B–B3)** of wild-type controls, Rab11a was located in the basal body of hair cells overlapping with the expression of γ-tubulin. The basal body was labeled with an antibody to γ-tubulin (red). The cytoskeleton was labeled with phalloidin (green), and staining for Rab11a is shown in blue. Scale bars: 10 μm.

Supplementary Figure 3 **(A,A1)** Normal appearance of anterior cristae in wild-type controls. The utricle is labeled with an anti-β-spectrin, the hair cell basal body is labeled with anti-γ-tubulin (blue), while stereocilia bundles are labeled with phalloidin (green). In the anterior and superior cristae of the mouse vestibular system, the well-organized planar cell coordination can be observed. **(B,B1)** In *Rab11a* mutants, the alignment of neighboring hair cells was not disrupted in P2 hair cells of the anterior cristae. **(C,C1)** Tissue planar cell polarity could be seen in hair cells from the lateral cristae of wild-type controls. **(D,D1)** In *Rab11a* mutants, all neighboring hair cells retained the same orientation. Scale bars: 50 μm.

Supplementary Figure 4 The *Rab11a*^*CKO/CKO*^ utricle **(A)** was labeled with anti-β-spectrin **(A1)**, the hair cell basal body **(A2)** like IFT88 was labeled with anti-γ-tubulin (blue), and stereocilia bundles **(A3)** were labeled with phalloidin (green).
