# Supplementary figures and images for: Rab11a Regulates the Development of Cilia and Establishment of Planar Cell Polarity in Mammalian Vestibular Hair Cells
Source: Front Mol Neurosci. 2021 Nov 19;14:762916. doi: 10.3389/fnmol.2021.762916 (PMC8640494; doi:10.3389/fnmol.2021.762916)

Sup Fig. 1

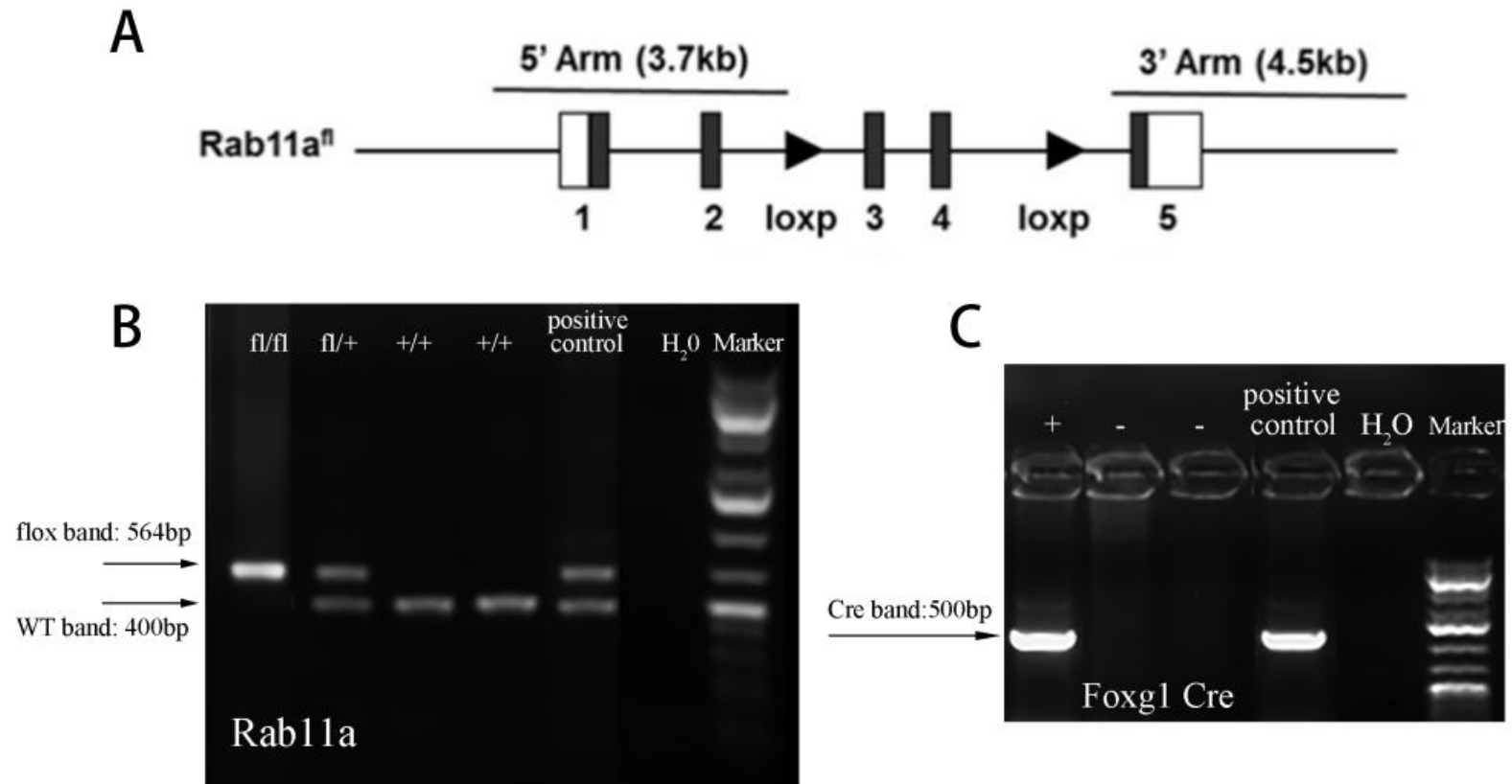

Sup Fig. 2

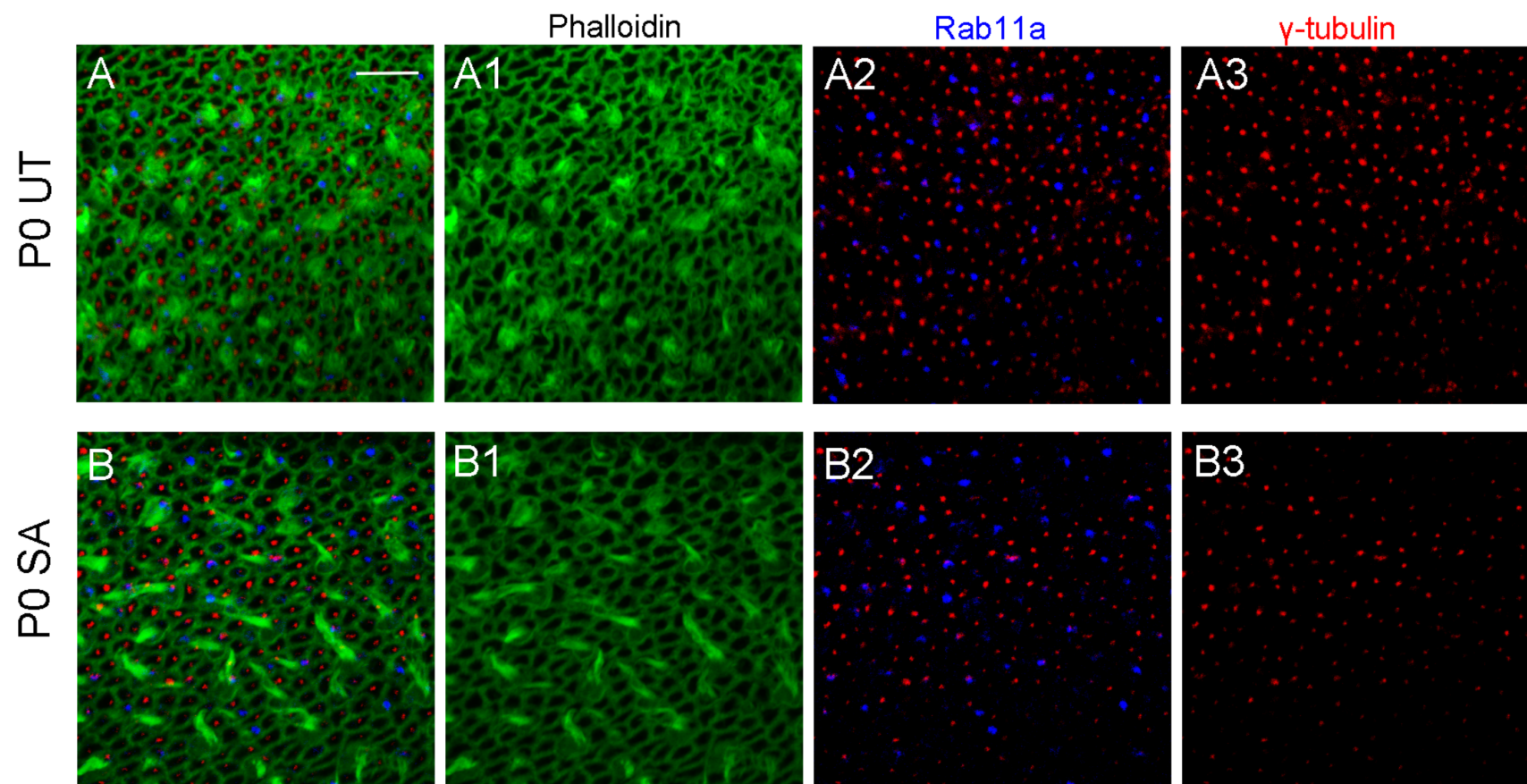

Sup Fig. 3

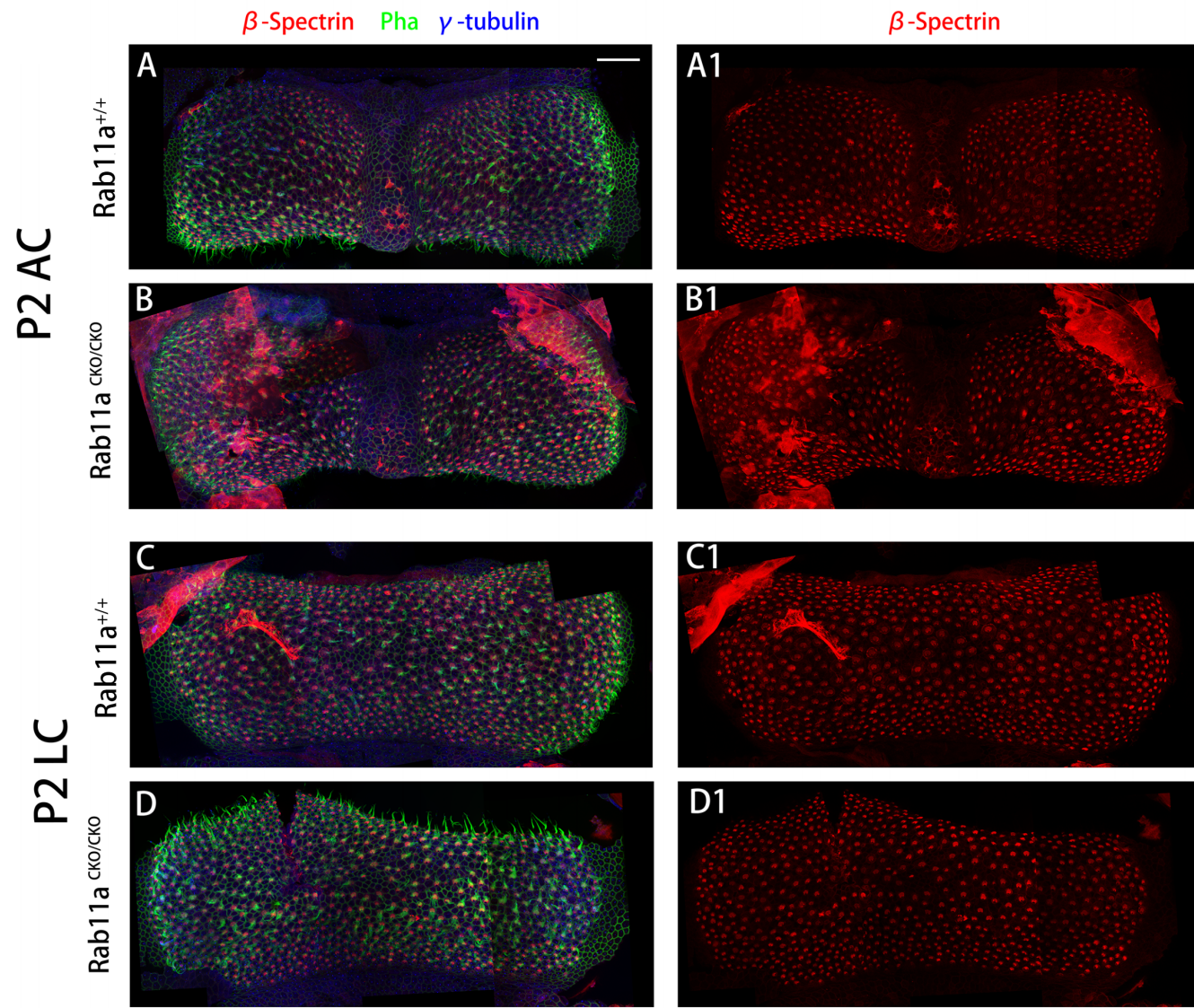

Sup Fig. 4

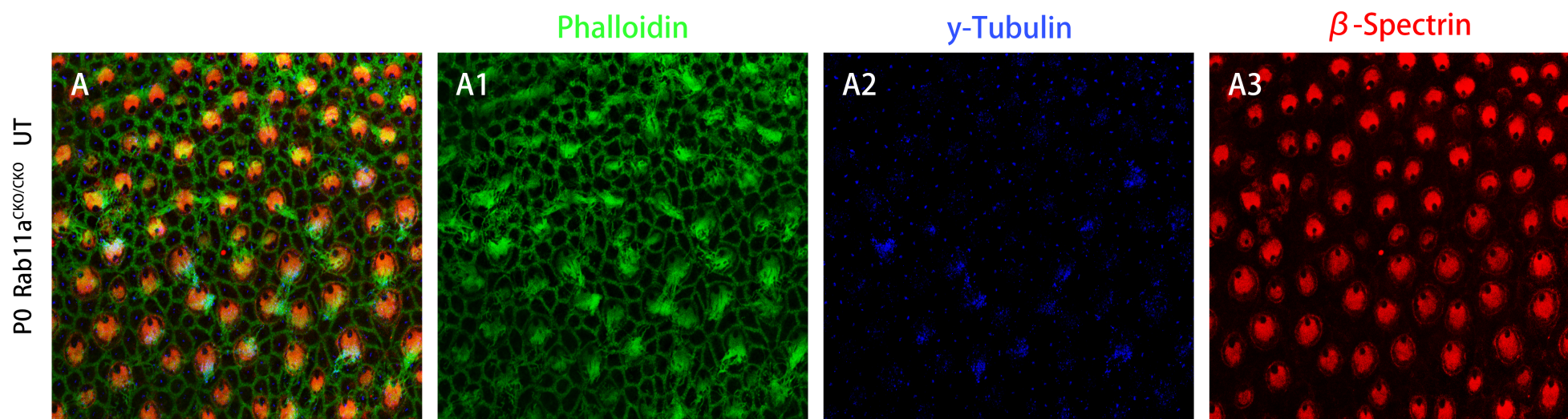

Supplement: Supplementary Figure 1 — (A) Rab11a gene conditional knockout mice were constructed by inserting two Loxp sites between exons 2 and 5, and excising specific sheared exons 3,4 by binding to Cre recombinase. (B) Identification of genotype of Rab11a: the genotype of Rab11fl/fl we need to obtain. Three cases can be seen in the figure. Pure type Rab11fl/fl has a flox band with a product size of 564 bp. wild type Rab11+/+ has a WT band with a product size of 400 bp. heterozygous Rab11a has both flox and WT bands. (C) Identification of genotype of Foxg1Cre: To specifically knock out Rab11a in the inner ear, Foxg1Cre+ was selected for specific expression. Cre-positive had a band and the product size was 500 bp. [file Image_1.pdf]
